# Evidence for abnormal linkage between urine oxalate and citrate excretion in human kidney stone formers

**DOI:** 10.14814/phy2.14943

**Published:** 2021-07-07

**Authors:** Megan L. Prochaska, Orson W. Moe, John R. Asplin, Fredric L. Coe, Elaine M. Worcester

**Affiliations:** ^1^ Department of Medicine University of Chicago Medicine Chicago IL USA; ^2^ Department of Internal Medicine University of Texas Southwestern Medical Center Dallas TX USA; ^3^ Litholink Corporation Laboratory Corporation of America® Holdings Itasca IL USA

**Keywords:** calcium oxalate, citrate, nephrolithiasis, oxalate

## Abstract

**Background:**

Animal models have demonstrated an interactive relationship between the epithelial anion exchanger SLC26A6 and transporter NaDC‐1 that regulates citrate and oxalate homeostasis. This relationship is a potential mechanism to protect against kidney stones as higher urine oxalate is accompanied by higher urine citrate but it has not been explored in humans.

**Methods:**

We examined 24‐h urine data on 13,155 kidney stone forming patients (SF) from separate datasets at the University of Chicago and Litholink, a national laboratory, and 143 non‐kidney stone forming participants (NSF) to examine this relationship in humans. We used multivariate linear regression models to examine the association between oxalate and citrate in all study participants and separately in SF and NSF.

**Results:**

Higher urinary oxalate was associated with higher urinary citrate in both SF and NSF. In NSF, the multivariate adjusted urine citrate excretion was 3.0 (1.5–4.6) (mmol)/creatinine (mmol) per oxalate (mmol)/creatinine (mmol). In SF, the multivariate adjusted urine citrate excretion was 0.3 (0.2–0.4) (mmol)/creatinine (mmol) per oxalate (mmol)/creatinine (mmol).

**Conclusions:**

Higher urinary oxalate excretion was associated with higher urinary citrate excretion and this effect was larger in non‐kidney stone forming participants compared with those who form kidney stones.

## Graphical abstract

Adult male offspring of a rat model of polycystic ovary syndrome is born small compared to controls, and as adults have lower body weights, with higher cholesterol and proteinuria. Although the male PCOS offspring have similar blood pressure as controls, they have an exaggerated pressor response to angiotensin II. These data suggest that male offspring of PCOS women may develop cardiometabolic disease as adults or with aging.

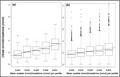

## 1 INTRODUCTION

High urine oxalate and low urine citrate are associated with higher risk of calcium oxalate kidney stone formation (Curhan & Taylor, 2008; Curhan et al., 2001). SLC26A6 is an epithelial anion exchanger that transports oxalate in the apical membrane of the renal proximal tubule and proximal intestine (Aronson, 2010; Jiang et al., 2006; Lee et al., 2012). NaDC‐1 is a sodium‐coupled dicarboxylate transporter that is also located in the apical membrane of the renal proximal tubule and small intestine (Pajor, 2006). Previous work in animal models demonstrated an interactive relationship between SLC26A6 and NaDC‐1 that regulates citrate and oxalate homeostasis (Ohana et al., 2013). NaDC1 activity is inhibited by SLC26A6 while SLC26A6 is activated by NaDC1 (Ohana et al., 2013). This reciprocal relationship suggests that when SLC26A6 is actively excreting oxalate in the urine it also inhibits NaDC1‐mediated reabsorption of citrate in the proximal tubule, leading to increased citrate excretion (Ohana et al., 2013).

Such a relationship may be an important protection against kidney stones because of a built‐in mechanism where higher urine oxalate would be accompanied by higher urine citrate, which is protective against calcium oxalate kidney stones. In the urine, citrate binds calcium to form a soluble calcium‐citrate complex instead of the insoluble calcium‐oxalate compound that eventually becomes a calcium oxalate kidney stone (Zacchia & Preisig, 2010). The biologic teleological reason for the activation of SLC26A6 by NaDC‐1 is less apparent. It is possible that some of these effects may be observed under special conditions *in vitro*. *In vivo* rodent data clearly showed that complete disruption of SLC26A6, which is a drastic maneuver, led to increased renal oxalate and decreased citrate levels (Ohana et al., 2013).

However, this homeostatic oxalate‐citrate relationship has not been explored in humans. It is possible that this oxalate‐citrate regulatory mechanism is disrupted in stone formers. Additionally, it is possible that an association between oxalate and citrate is noted in humans and that the mechanism for this association is driven by consumption of foods that contain both oxalate and potassium alkali, raising urine citrate with oxalate. We hypothesized that this mechanism is present in humans, and that there is a pathologic disruption in kidney stone forming patients so that the protective mechanism of higher citrate excretion associated with higher oxalate excretion may be blunted.

## 2 METHODS AND METHODS

### 2.1 Subjects

Subjects were selected from three populations with 24‐h urine data. The study was approved by the IRB at the University of Chicago (IRB # 11943A) and by Western IRB protocol # 20162248 (Litholink).

#### 2.1.1 Non‐Stone forming participants

Non‐stone formers (NSF) were selected from the University of Chicago Kidney Stone Research Program registry. We selected individuals who have no personal or family history of kidney stones and were not on medications. Each subject completed at least one 24‐h urine collection. For NSF participants with more than one 24‐h urine collection, the mean value of the collections was used for each urinary variable. Our final dataset included 77 males and 66 females.

#### 2.1.2 Stone formers

Stone formers (SF) were selected from the University of Chicago Kidney Stone Evaluation and Treatment Program (UCM SF), which has its own clinical laboratory, and from Litholink (LabCorp), a national kidney stone testing service (Litholink SF). We excluded patients with bowel disease, primary hyperparathyroidism, renal tubular acidosis and cystinuria. At Litholink these exclusions were via intake telephone interviews, at the University of Chicago they were based on chart review. Primary hyperparathyroidism and renal tubular acidosis exclusions were also made based on presence of hypercalcemia, or the combination of low total serum bicarbonate and potassium, respectively.

From the University of Chicago we selected idiopathic calcium stone formers who completed three 24‐h urine collections with matching fasting blood sample prior to treatment between 1970 and 2008. At the time of the collection subjects were off all medications that could affect stone formation or mineral metabolism. In addition to exclusions noted above, we were able to exclude patients with sarcoidosis, vitamin D excess, and stones containing any struvite. Our final dataset included 414 males and 200 females.

Litholink SF collected one complete 24‐h collection prior to treatment of kidney stones. These patients were instructed to refrain from taking vitamin C, but compliance could not be verified. The initial dataset included 12,839 participants, the final data set after exclusions included 7,103 males and 5,438 females.

### 2.2 Serum and urine data

The University of Chicago and Litholink laboratories have exchanged urine samples since the founding of Litholink in 1995, ensuring comparability of results (Bergsland et al., 2018). Twenty‐four‐hour urine data included volume, creatinine, calcium, oxalate, citrate, potassium, uric acid, pH, chloride, phosphorus, magnesium, ammonium, and sulfate. Measurements were made using methods detailed elsewhere (Bushinsky et al., 2002).

From all data sets we excluded participants with missing data, urine creatinine <500 or >3000, urine citrate <30 mg/day, urine ammonia excretion >100 mmol/day or age <18. Exclusion for urine citrate <30 mg/day were made as this is the limit of detectability of the assay.

### 2.3 Statistical analysis

Mean and standard deviations were calculated for all urine variables. Urine gastrointestinal alkali absorption (GI anion) was calculated by subtracting non‐combustible anions from non‐combustible cations [(Na + K + Ca + Mg) – (Cl + 1.8*P)], with all analytes in meq/liter except phosphorus which is in mmol/liter; multiplication by 1.8 converts P to meq at the pH of blood (Oh, 1989). Urinary excretion of both oxalate and citrate were converted to mmol per mmol creatinine for statistical analysis.

Univariate and multivariate linear regression were used to examine the association between 24‐h urinary oxalate excretion and 24‐h urinary citrate excretion for all study participants and in an analysis stratified by being a kidney stone forming patient. Multivariate models were adjusted for sex and 24‐h excretion of urine GI anion divided by creatinine (mmol). GI anion was included in the multivariate model to account for alkali intake. In the multivariate analysis including all participants, an interaction term with oxalate (mmol) per creatinine (mmol) by SF was included to evaluate for effect modification by being an SF. Oxalate mmol per creatinine mmol was divided into pentiles for both NSF and SF to generate boxplots of citrate mmol per creatinine mmol by oxalate pentile, and a trend analysis was done across the pentiles for each group.

In order to detect possible artifact from in vitro conversion of ascorbate to oxalate (), we performed a sub‐analysis limiting urine pH to <6.5. Because this did not alter the conclusions of the paper, we do not present the results.

All statistical analyses were performed using R.

## 3 RESULTS

The 24‐h urine characteristics of the 143 NSF, 614 UCM SF and 12,541 Litholink SF are shown in Table 1. As expected, mean urinary calcium was higher in SF than in NSF, as was sodium, and calcium oxalate, calcium phosphate and uric acid supersaturations. Although urinary oxalate levels are higher in SF than NSF, urine citrate levels did not differ (Table 1).

**TABLE 1:** Characteristics of patients in datasets

Variable	NSF (*N* = 143)	UCM SF (*N* = 614)	Litholink SF (*N* = 12,541)
Age (years)		45 (12)	48 (15)
Male N, %	77 (54%)	414 (67%)	7,103 (57%)
Volume (L/d)	1.7 (0.9)	1.8 (0.8)	1.8 (0.8)
Calcium (mg/d)	161 (95)	242 (112)^a^	226 (118)^c^
Sodium (mEq/d)	151 (68)	179 (62)^a^	189 (77)^c^
Oxalate (mg/d)	36 (15)	43 (15)^a^	39 (16)^d^
Magnesium (mg/d)	102 (45)	107 (36)	104 (41)
Citrate (mg/d)	566 (283)	555 (286)	606 (321)
Potassium (mEq/d)	64 (25)	61 (22)	61 (25)
Sulfate (mEq/d)	49 (23)	46 (18)	43 (18)^c^
Phosphorus (mg/d)	872 (324)	982 (293)^a^	1011 (376)^c^
NH4 (mmol/day)	32 (16)	32 (19)	38 (15)^c^
Creatinine (mg/d)	1442 (474)	1690 (443)^a^	1613 (514)^c^
Chloride (mmol/d)	149 (63)	171 (60)^a^	177 (72)^c^
pH	6.1 (0.4)	6.0 (0.4)^b^	6.0 (0.5)^d^
Calcium oxalate supersaturation	6.1 (3.5)	9.3 (4.0)^a^	7.7 (3.9)^c^
Calcium phosphate supersaturation	1.1 (0.8)	1.5 (0.9)^a^	1.4 (1.1)^c^
Uric Acid mg/day	594 (202)	671 (195)^a^	684 (230)^c^
GI anion (meq/d)	31 (23)	33 (23)	35 (25)

Presented as mean (standard deviation) unless noted.

NSF, non‐kidney stone forming participants; SF, stone forming participants

^a^ <0.001 for UCM SF compared to NSF.

^b^ <0.05 for UCM SF compared to NSF.

^c^ <0.001 for Litholink SF compared to NSF.

^d^ <0.05 for Litholink SF compared to NSF.

**FIGURE 1:**
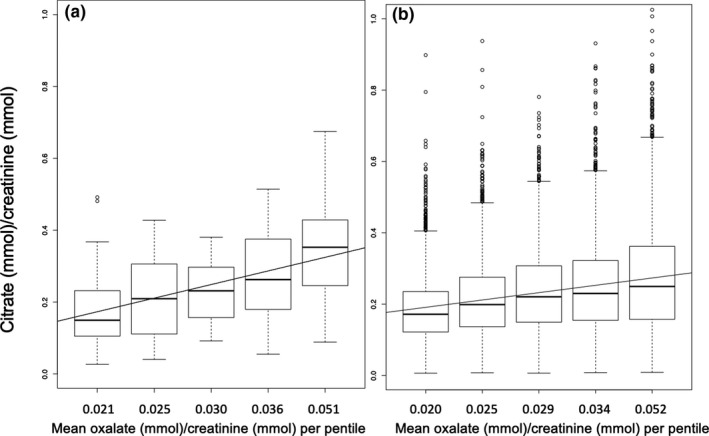
Urine citrate (mmol)/creatinine (mmol) by pentiles of urine oxalate (mmol)/ creatinine (mmol). Panel (a): Non‐kidney stone formers (NSF). Panel (b): Kidney stone formers (SF). Boxplots were created by generating 5 equal groups based on level of oxalate (mmol)/creatinine (mmol) for both NSF and SF. Mean oxalate for each pentile shown on the X‐axis. Range of oxalate (mmol)/creatinine (mmol) per pentiles: NSF ‐ 1: 0.0168–0.0231, 2: 0.0232–0.0274, 3: 0.0275–0.0327, 4:0.0328–0.0404, 5:0.0405–0.0813. SF ‐ 1: 0.0073–0.0229, 2: 0.0229–0.0269, 3:0.0269–0.0314, 4:0.0314–0.0384, 5:0.0384–0.5628. Citrate (mmol)/creatinine (mmol) levels are displayed with upper and lower extremes, interquartile range (box), with median (center line), and outliers (open circles) per oxalate (mmol)/creatinine (mmol) pentile. Line demonstrates linear regression line for citrate/creatinine by oxalate/creatinine pentiles. P for trend <0.001 for both NSF and SF

Level of urinary citrate rose progressively with increasing pentiles of oxalate excretion (Figure 1). The slope of the line for NSF was 0.04 compared with 0.02 for SF, *p* < 0.001 for trend for both.

### 3.1 Stratified multivariable models

#### 3.1.1 NSF

In an analysis stratified by SF status, higher oxalate was associated with higher citrate for 143 NSF with the multivariable regression slope of 3.0 (1.5–4.6) citrate (mmol)/ creatinine (mmol) per oxalate (mmol)/ creatinine (mmol) (Table 2), highly compatible with the results from the multivariable model including NSF and SF.

**TABLE 2:** Models for prediction of change in citrate (mmol)/creatinine (mmol) per change in oxalate (mmol)/creatinine (mmol) in non‐kidney stone forming participants and stone forming participants

Model	Change in (citrate/creatinine) per (oxalate/creatinine) (95% CI) for NSF (N=143)	*p*	Change in (citrate/creatinine) per (oxalate/creatinine) (95% CI) for SF (N=13,155)	*p*
Univariate	5.3 (3.6–6.9)	<0.001	1.8 (1.6–1.9)	<0.001
MV model	3.0 (1.5–4.6)	<0.001	0.3 (0.2–0.4)	<0.001

Abbreviations: CI, confidence intervals; MV, multivariate model; NSF, non‐kidney stone forming participants; SF, stone forming participants.

^a^ MV model: urine GI anion (meq)/ creatinine (mmol), sex.

^b^ citrate, oxalate, and creatinine all in mmol.

#### 3.1.2 SF

Among only SF (*N *= 13,155), the regression slope in the multivariable model was 0.3 (0.2–0.4) citrate (mmol)/ creatinine (mmol) per oxalate (mmol)/ creatinine (mmol) (Table 2), compatible with the overall model. In separate analyses within the two SF patient datasets results were overall similar (Table S1).

### 3.2 Multivariable model for all participants combined

In an analysis including all 13,298 participants, higher urinary oxalate was associated with higher urinary citrate (Table 3). Likewise higher GI anion was associated with higher citrate. Female sex was associated with higher citrate levels (Table 3). The relationship between urinary citrate and oxalate was strongly influenced by kidney stone former status. The magnitude of the association was lower by 2.8 for SF compared with that in NSF (Table 3).

**TABLE 3:** Multivariate model to predict urine citrate (mmol)/creatinine (mmol) (*N* = 13,298)

Variable	Point estimate (95% CI)	*p*
Oxalate (mmol) / Creatinine (mmol)	3.1 (1.5–4.7)	<0.001
GI anion (meq) / Creatinine (mmol)	0.022 (0.021–0.023)	<0.001
Sex (female)	0.071 (0.67–0.075)	<0.001
Kidney stone former	0.08 (0.02–0.1)	0.001
Oxalate/Creatinine * kidney stone former	−2.8	<0.001

CI, confidence intervals.

Multivariate model including all study participants with citrate (mmol), oxalate (mmol), and GI anion (meq) all corrected for urine creatinine by dividing by creatinine (mmol). Interaction term was included for oxalate (mmol)/creatinine (mmol) by SF to show the effect modification of the oxalate and citrate association by being a kidney stone former.

## 4 DISCUSSION

In our study in humans, we found an association between oxalate and citrate in humans such that higher urine oxalate was association with higher urine citrate and the magnitude of the association was higher in NSF compared with SF. There are two possible mechanisms that may be contributing to this association and given the observational nature of this study we are unable to distinguish between these two mechanisms or why there is a difference between NSF and SF. The first mechanism is diet related and due to simultaneous consumption of oxalate and potassium alkali and the difference between NSF and SF may be due to differences in acid‐base and citrate handling between these two groups. The second possible mechanism is that this is related to a similar SLC26A6‐NaDC‐1 linkage that has been demonstrated in animal and cell models and additional study is needed to determine the mechanism for the difference between NSF and SF.

The risk of calcium oxalate kidney stone formation increases directly with rising urine oxalate excretion (Curhan & Taylor, 2008), likely consequent to the increase in calcium oxalate supersaturation that results (Prochaska et al., 2018). Urinary supersaturation with calcium oxalate promotes the formation and growth of crystals in the inner medulla and renal pelvis, especially at very high oxalate excretion levels, which can result in renal damage (Waikar et al., 2019) as well as kidney stones. However, urine contains inhibitors of crystallization that protect against crystal deposition in the kidney. The best understood of these is citrate, a tricarboxylic acid and intermediary metabolite in the citric acid cycle. Citrate binds urine calcium and thereby inhibits formation of poorly soluble calcium oxalate complexes (Zacchia & Preisig, 2010). In addition, citrate acts at the surface of formed crystals to inhibit their growth (Chung et al., 2016). Increasing urine citrate is associated with decreased risk of stone formation in epidemiologic studies (Curhan & Taylor, 2008).

Plasma citrate is filtered at the glomerulus, and reabsorbed in the proximal tubule by the luminal sodium‐dicarboxylate co‐transporter NaDC‐1. Since no further citrate transport occurs in the more distal nephron segments, proximal tubule reabsorption determines the citrate that ends up in the final urine. The amount of filtered citrate reabsorbed is primarily regulated by acid‐base status, under the control of the endothelin receptor (Liu et al., 2010), and requiring Pyk2 and ERK1/2 signaling pathways (Zacchia et al., 2018). However, recent work in animals and transfected cells suggests that a second mechanism may influence citrate absorption: interplay between NaDC‐1 and the proximal tubule oxalate‐transporting anion exchanger SLC26A6, also in the luminal membrane of the proximal tubule.

When co‐expressed in *Xenopus* oocytes, the activity of SLC26A6 affects the function of NaDC‐1, mediated by interactions between E613 in the STAS domain of SLC26A6 and K107 in the first intracellular loop of NaDC‐1 (Khamaysi et al., 2019); an interaction regulated by the scaffold protein IRBIT (IP_3_ receptor‐binding protein released with IP_3_) (Khamaysi et al., 2019). Increased transport of oxalate via SLC26A6 inhibited citrate transport by NaDC‐1 in this cellular model and the hyperoxaluria in the SLC26A6‐/‐ mice is accompanied by hypocitraturia (Khamaysi et al., 2019; Ohana et al., 2013).

The only exploration in humans thus far consisted of a calcium oxalate stone‐former who is a heterozygous carrier of the SLC26A6 polymorphism of SLC26A6(D23H/D673N) which is characterized by reduced expression and function of SLC26A6 when studied in vitro. Importantly, this SLC26A6 variant does not fully suppress NaDC‐1 resulting in hypocitraturia that is not explained by increased acid load (Shimshilashvili, 2020).

If the above proposed model were true in humans, it can represent a biologic mechanism endowed by nature to increase urine citrate to offset an increase in urine oxalate, thereby decreasing the risk of stone formation. Furthermore, the ability of stone formers to augment citrate excretion as oxalate excretion increases may be impaired thus predisposing them to calcium oxalate precipitation.

This study is the first to explore the implications of this work in humans, both SF and NSF, using large data sets of 24‐h urine collections from both. Two important findings emerged. First, we found a significant relationship between oxalate and citrate excretion, such that higher urine oxalate was associated with higher urine citrate in both SF and NSF. Second, the magnitude of the effect was significantly greater in non‐stone forming participants, with higher urine citrate at a given oxalate excretion. Sex and GI alkali absorption were significant covariates in the model. The weakened effect of high urine oxalate on urine citrate may contribute to the stone risk in the stone formers.

Our study has limitations. This is an observational study. We did not have clinical data for Litholink SF, and we only had one urine collection per participant for Litholink SF. We did not have data on age for NSF. We do not have dietary data for any of the study participants and thus cannot evaluate the effect of diet on these observations. Foods that are high in oxalate may also be higher in citrate and we are unable to evaluate the contribution of consumption of such foods to these results. However, our models included the effects of GI anion which is the gauge of alkali absorption from foods, so that in part this limitation has been addressed. In addition, the UCM patients have been selected to represent idiopathic calcium stone formers, while the Litholink patients may be less uniform. This heterogeneity would tend to weaken our findings by increasing variability in the results.

In conclusion, in this large study of kidney stone forming and non‐kidney stone forming human participants, we found that higher urinary oxalate was associated with higher urinary citrate and that this effect was larger in non‐kidney stone forming participants compared with those who form stones. Additional study is needed to test potential mechanisms for the association.

## CONTRIBUTIONS

Prochaska: project design, data analysis and interpretation, manuscript writing and revision; Moe: project conception, manuscript revision; Asplin: data collection, manuscript revision; Coe: project conception, data interpretation, manuscript revision; Worcester: project conception, data collection, manuscript writing and revision. All authors have given final approval to the manuscript.

## CONFLICT OF INTEREST

The authors declare that they have no conflicts of interest with this work.

## Supporting information



Table S1
