# Supplementary material for: Evidence for abnormal linkage between urine oxalate and citrate excretion in human kidney stone formers
Source: Physiol Rep. 2021 Jul 7;9(13):e14943. doi: 10.14814/phy2.14943 (PMC9814525; doi:10.14814/phy2.14943)
Supplement: Supplementary file 1 — Table S1 [file PHY2-9-e14943-s001.docx]

**Supplemental Table 1**. Models for prediction of change in citrate (mmol)/creatinine (mmol) per change in oxalate (mmol)/creatinine (mmol) for stone formers (SF) in Litholink (N=12,541) and University of Chicago (UCM) (N=614)

| Model | Change in (citrate/creatinine) per (oxalate/creatinine) (95% CI) for Litholink SF (N=12,541) | p | Change in (citrate/creatinine) per (oxalate/creatinine) (95% CI) for UCM SF (N=614) | p |
| --- | --- | --- | --- | --- |
| univariate | 1.8 (1.6 to 1.9) | <0.001 | 2.3 (1.5 to 3.1) | <0.001 |
| MV model | 0.3 (0.1 to 0.4) | <0.001 | 1.0 (0.2 to 1.7) | 0.02 |

* MV model: urine GI anion (meq)/ creatinine (mmol), sex

Abbreviations: NSF, non-kidney stone forming participants; SF, stone forming participants; MV, multivariate model; CI, confidence intervals; UCM, University of Chicago Medicine
